# Supplementary material for: Motility and microtubule depolymerization mechanisms of the Kinesin-8 motor, KIF19A
Source: eLife. 2016 Sep 30;5:e18101. doi: 10.7554/eLife.18101 (PMC5045296; doi:10.7554/eLife.18101)
Supplement: Supplementary file 1. — Data collection and refinement statistic table. DOI: http://dx.doi.org/10.7554/eLife.18101.025 [file elife-18101-supp1.docx]

**Table S1.** **Data Collection and Refinement Statistics**

|  | KIF19A-353WT-ADP |
| --- | --- |
| **Data collection** | SPring-8 BL41XU |
| Space group | P4_2_2_1_2 |
| Cell dimensions |  |
| *a*, *b*, *c* (Å) | 122.604, 122.604, 56.198 |
| α, β, γ (°) | 90, 90, 90 |
| Resolution (Å) | 50-2.718(2.81-2.71)^*^ |
| *R_sym_ or R*_merge_ | 0.132(0.995) |
| *I* / σ*I* | 7.5(0.1) |
| Completeness (%) | 99.9(99.3) |
| Redundancy | 13.6(9.2) |
|  |  |
| **Refinement** |  |
| Resolution (Å) | 34.3-2.718 |
| No. reflections | 12,043 |
| *R*_work_ / *R*_free_ | 0.222/0.302 |
| No. atoms |  |
| Protein | 2,243 |
| Ligand/ion | 40 |
| Water | 28 |
| *B*-factors (Å^2^) | 57.1 |
| R.m.s. deviations |  |
| Bond lengths (Å) | 0.008 |
| Bond angles (°) | 1.170 |

* Values in parentheses are for highest resolution shell.
